# Oxytocin as an adolescent treatment for methamphetamine addiction after early life stress in male and female rats

**DOI:** 10.1038/s41386-022-01336-y

**Published:** 2022-05-17

**Authors:** Sarah J. Baracz, Katherine J. Robinson, Amanda L. Wright, Anita J. Turner, Iain S. McGregor, Jennifer L. Cornish, Nicholas A. Everett

**Affiliations:** 1 School of Psychological Sciences, Macquarie University, North Ryde, NSW 2109 Australia; 2 School of Psychology, University of Sydney, Camperdown, NSW 2006 Australia; 3 Centre for Emotional Health, Macquarie University, North Ryde, NSW 2109 Australia; 4 Centre for Motor Neuron Disease Research, Macquarie Medical School, Macquarie University, North Ryde, NSW 2109 Australia; 5 Lambert Initiative of Cannabinoid Therapeutics, Brain and Mind Centre, University of Sydney, Camperdown, NSW 2050 Australia

**Keywords:** Stress and resilience, Addiction

## Abstract

Early life stress (ELS) is associated with perturbed neural development and augmented vulnerability to mental health disorders, including addiction. How ELS changes the brain to increase addiction risk is poorly understood, and there are no therapies which target this ELS-induced vulnerability. ELS disrupts the oxytocin system, which can modulate addiction susceptibility, suggesting that targeting the oxytocin system may be therapeutic in this ELS-addiction comorbidity. Therefore, we determined whether adolescent oxytocin treatment after ELS could: (1) reduce vulnerability to anxiety, social deficits, and methamphetamine-taking and reinstatement; and (2) restore hypothalamic oxytocin and corticotropin-releasing factor expressing neurons and peripheral oxytocin and corticosterone levels. Long Evans pups underwent maternal separation (MS) for either 15 min or 360 min on postnatal days (PND) 1–21. During adolescence (PNDs 28–42), rats received a daily injection of either oxytocin or saline. In Experiment 1, adult rats were assessed using the elevated plus-maze, social interaction procedure, and methamphetamine self-administration procedure, including extinction, and cue-, methamphetamine- and yohimbine-induced reinstatement. In Experiment 2, plasma for enzyme immunoassays and brain tissue for immunofluorescence were collected from adult rats after acute stress exposure. Adolescent oxytocin treatment ameliorated ELS-induced anxiety and reduced methamphetamine- and yohimbine-induced reinstatement in both sexes, and suppressed methamphetamine intake and facilitated extinction in males only. Additionally, adolescent oxytocin treatment after ELS restored oxytocin-immunoreactive cells and stress-induced oxytocin levels in males, and attenuated stress-induced corticosterone levels in both sexes. Adolescent oxytocin treatment reverses some of the ELS effects on later-life psychopathology and vulnerability to addiction.

## Introduction

Stress exposure during childhood is associated with increased risk of mental illness in adulthood, including depression, anxiety, and aggressive behaviour [1]. Childhood trauma also drives higher rates of drug abuse and younger age of initiating drug-taking behaviour, including use of the highly addictive psychostimulant methamphetamine [2–4]. Conservative estimates indicate that 8.9 per 1000 children in the USA experience childhood trauma [5], suggesting that many individuals are vulnerable to mental health problems due to adverse early life experiences.

Exposure to early life stress (ELS) can also disturb the normal trajectory of neural development. Neuroendocrine systems involving the neuropeptide oxytocin are strongly impacted. ELS changes oxytocin and oxytocin receptor expression [6, 7], which disturbs oxytocin system functions, including modulation of stress responses, emotions, experiences of reward, and social functioning [8]. Moreover, oxytocin system disruption induced by ELS is associated with a greater susceptibility to drug addiction [8, 9]. Considering this critical functional role of oxytocin, rebalancing the oxytocin system through exogenous administration of oxytocin may ameliorate psychopathology linked to ELS.

Previous work by our group and others has explored the efficacy of oxytocin in reversing phenotypic abnormalities in animals with a history of ELS. Acute [10] and chronic [11] oxytocin administration in adult male rodents reduced depression-like behaviour induced by ELS while oxytocin administered in adolescence *prevented* ELS-induced depression-like behaviour in adult male and female rats [12]. Since adolescence is a neurodevelopmental window whereby brain systems can be recalibrated after ELS [13], it is conceivable that oxytocin treatment during adolescence rebalances the ELS-induced dysregulation of the oxytocin system, preventing associated psychopathology. However, this hypothesis remains speculative.

The primary aim of the present study was to determine whether adolescent oxytocin treatment prevents ELS-induced vulnerability to METH-taking and reinstatement, anxiety, and social deficits. To interrogate the possible neurobiological interactions between oxytocin, ELS, and METH use, we also investigated whether adolescent oxytocin treatment reverses ELS-induced perturbations of the central and peripheral oxytocin system, and the HPA-axis.

## Materials and Methods

### Animals

15 time-mated Long Evans dams (purchased from Animal Resource Centre, Perth) arrived on gestation day 17. Dams were housed individually (21 °C ± 1 °C) with *ad libitum* food and water. Experiments occurred during the light cycle (on 07:00, off 19:00), were conducted in accordance with the Australian Code of Practice for the Care and Use of Animals for Scientific Purposes (8^th^ edition, 2013), and were approved by the Macquarie University Animal Ethics Committee. It should be noted that dams may have experienced an additional source of maternal stress on gestational day 15–16 as they were transported to our facility, although dams in the control and maternal separation conditions were equally exposed to this.

### Drugs

Methamphetamine hydrochloride (METH) was purchased from the Australian Government Analytical Laboratories (Australia), oxytocin from China Peptides (China) and yohimbine hydrochloride from Tocris Bioscience (United Kingdom). For intraperitoneal (i.p.) injections, METH was injected at a volume of 1 ml/kg, whilst oxytocin and yohimbine were injected at 2 ml/kg.

### Maternal separation procedure

Maternal separation (MS) was conducted identically to an established protocol [12, 14, 15]. Pups were cross-fostered on postnatal day (PND) 0 to produce equivalent-sized litters, with a similar ratio of male to female pups (approximately 50–60% male) in each litter. Litters were randomly allocated to be separated from the dam as a litter for either 15 (MS15) or 360 min (MS360) per day from PND 1–21. This separation was conducted at the same time each day (09:30–9:45 for MS15 condition and 10:00–16:00 for the MS360 condition). MS15 is extensively implemented as a control condition in MS studies [12, 14–18]. Rats were weaned on PND 22 into cages of 2–3 of the same sex and litter.

### Adolescent drug treatment

**Fig. 1:**
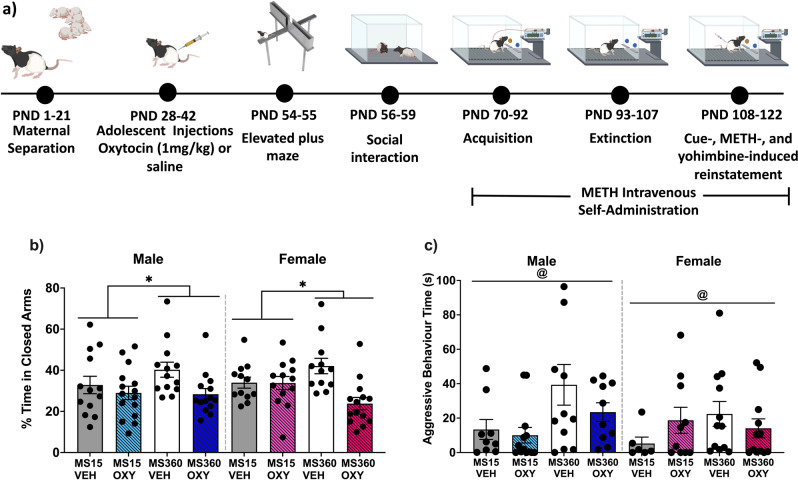
Effect of early life stress and adolescent oxytocin treatment on anxiety-like behaviour and social aggression. **a** Experimental timeline depicting behavioural testing in male and female Long Evans rats after ELS and adolescent oxytocin injections. This figure was created using BioRender (academic subscription). ELS = early life stress, METH = methamphetamine, VEH = vehicle, OXY = oxytocin. **b** Maternal separation (MS360) increased anxiety-like behaviour relative to separation controls (MS15), which was prevented by adolescent oxytocin treatment in males and females. Percentage (± SEM) of time spent in the closed arms of the EPM by condition (*n* = 12–15/condition/sex). **c** Maternal separation (MS360) increased aggressive behaviour towards a novel conspecific relative to separation controls (MS15). Mean (± SEM) duration spent engaging in aggressive behaviour by condition (*n* = 6–12 pairs/condition/sex). @*p* < 0.05 significant MS effect. *p < 0.05 significant interaction effect.

Rats were randomly assigned to receive either i.p. vehicle (saline) or oxytocin (1 mg/kg) daily from PND 28–42 (early to mid-adolescence in rats [19]). Treatment allocation included matching for weight. This dose was based on previous research by our group [12, 20, 21] and others [22]. From PND43, rats were left undisturbed until testing commenced (Figs. 1a and 4a).

## Experiment 1

### Elevated plus maze

During early adulthood (PND 54–55), anxiety-like behaviour was measured using the elevated plus maze [14]. Briefly, the elevated plus maze was elevated 50 cm off the floor in a dark room, with open and closed arms measuring 50 cm long × 10 cm wide, and the closed arms having 40 cm high walls. The arena was illuminated from above with a white light (40 lux), and video recording was conducted with an infrared camera. Rats were placed on the apparatus and the experimenter immediately left the room for 5-min. The apparatus was cleaned with 70% ethanol and dried between rats. Percentage of time spent in the closed arms relative to the total 5-min was analysed using CleverSys software (USA).

### Social interaction

Social interaction testing was conducted in black arenas (50 × 50 × 50 cm) with dim red lighting [21]. On PND 56, rats were individually habituated to the arena for 20-min. On PND 57 and 59, rats were matched with an unfamiliar conspecific of the same sex, separation and treatment condition, and a similar weight (±10%). Each test involved a novel pairing of unfamiliar rats. On each day, the rat pairs were placed in the arena and their behaviour was recorded for 10-min and scored using ODLog (http://www.macropodsoftware.com/odlog/). Rats had a day off from testing (PND 58) where they were left undisturbed in their home cages. As we do not see an effect of day in this procedure, each dyadic interaction was considered an individual datapoint.

Behaviours included: anogenital sniffing, general investigation (sniffing of the conspecific), and aggressive behaviours (pinning, chasing, tumbling, and mounting of the conspecific). Aggressive behaviours typically occurred around the onset of a mutual-upright event, a well studied aggressive posture, and were accompanied by piloerection, tail rattling, and lateral orientation, which can differentiate play from aggression [23].

### Intravenous catheter surgery

On PND60, rats underwent catheterization surgery followed by post-operative care, as previously described [24]. Briefly, rats were anesthetized with 3% isoflurane, and a custom-made catheter made of silastic tubing was inserted into the right jugular vein. The catheter was connected to a right-angled steel cannula externalized 2 cm caudal to the scapula, which connected to the drug infusion pump during self-administration sessions, or sealed with plastic and brass screw caps. The catheter was flushed daily with a 0.2 ml saline solution containing heparin (60 IU) and cephazolin (20 mg).

### Acquisition and maintenance of intravenous self-administration

Rats acquired intravenous self-administration (IVSA) of METH during daily 2-h fixed ratio-1 scheduled sessions in standard operant response chambers (Med Associates, USA; [8]). Lever extension and house light illumination indicated the initiation of the session and METH availability. Depression of the active lever delivered a 3-s infusion of METH, cue light illumination, house light termination, and a 20-s time-out period, during which pressing the active lever had no programmed consequences. Rats first self-administered a low dose of METH (0.03 mg/kg/infusion) for 10 days, and then a higher dose (0.1 mg/kg/infusion) for 12 days. Intake was limited to 120 infusions (day 1–10) and 60 infusions (day 11–22) per session.

### Extinction

During extinction sessions (2 h/day), depression of the active lever had no programmed consequences, and the house light remained off. Extinction continued for a minimum of 14 days. Rats met extinction criteria when <15 lever presses were made over three consecutive sessions.

### Reinstatement

Rats underwent testing for cue-, METH- and yohimbine-induced reinstatement (2 h). These tests were separated by 2–3 extinction sessions to ensure that rats reached extinction criteria (<15 presses) before proceeding to the next reinstatement test. For cue-induced reinstatement (1 h), rats were re-exposed to METH-associated cues, whereby the house light illuminated with the initiation of the session, and the cue light illuminated (3 s) upon active lever press. For METH-primed reinstatement, rats underwent 3 tests, where they received vehicle (saline), 0.3 or 1 mg/kg METH i.p. immediately prior to an extinction session. For yohimbine-induced reinstatement, rats were injected with the pharmacological stressor yohimbine, an α2 adrenoceptor antagonist, which elicits reinstatement to drug-seeking in rats and cravings in humans [25, 26]. Rats received vehicle (distilled water), 0.625 or 1.25 mg/kg yohimbine i.p. 30 min prior to an extinction session. For all METH- and yohimbine-primed reinstatement tests, rats were placed in the operant chamber for 1 h, with identical programming as the extinction sessions. Reinstatement tests were conducted sequentially, beginning with cue-, then METH (0.3 then 0.1 mg/kg) and yohimbine-induced reinstatement (0.625 then 1.25 mg/kg).

## Experiment 2

### Acute stressor

In early adulthood (PND 60–65), rats were exposed to an acute stressor, a brightly lit novel open field (100 × 100 × 60 cm, ~900 lux). Each rat was placed in the centre of the apparatus for 10-min, and then individually housed for 2 h until sacrifice [27–29].

### Enzyme-linked immunosorbent assays, immunofluorescence and microscopy

Cardiac blood and brain tissue was extracted using published methods [30]. Briefly, rats were anesthetized with an overdose of sodium pentobarbitone (135 mg in 1 ml, i.p.), cardiac blood was collected using a 23 g needle and syringe, placed into heparinized tubes on ice, centrifuged (1600 g, 15 min at 4 °C), and the plasma supernatant was aliquoted and stored at −80 °C. A transcardial perfusion was then performed (300 ml Dulbecco’s Modified Eagle Medium (Sigma-Aldrich) followed by 300 ml fresh ice cold 4% paraformaldehyde, at 16 ml/min). Brains were post-fixed in 4% paraformaldehyde for 24 h at 4 °C, and then placed in a cryoprotectant solution (20% sucrose, 30% ethylene glycol, 2% polyvinylpyrrolidone dissolved in 0.1 mol PBS) for storage at −20 °C until slicing.

Plasma levels of oxytocin and corticosterone were assayed using ELISA kits (ENZO Life Sciences) and normalized to total protein concentrations determined via BCA assay [31] (Thermo Fisher). Oxytocin samples were extracted using solid-phase extraction.

Brains were sectioned at 50 μm (coronal) and immunohistochemically stained using an established protocol [14, 30]. The antibodies used were rabbit anti-oxytocin (1:1000, AB911, Merck Millipore, lot number: 2788898; RRID:AB_2157629) with donkey anti-rabbit-CY3, (1:500, A-31572 Thermo Fisher, lot number: 1837922; RRID:AB_162543) and guinea pig anti-corticotropin-releasing factor (CRF) (1:2000, T-5007, Peninsula Laboratories, lot number: A16758; RRID:AB_518256) with donkey anti-guinea pig-488 (1:500, 706–545–148, Jackson Immunoresearch, lot number: 134611; RRID:AB_2340472). Negative control, pre-adsorption and cross-reactivity experiments were performed to confirm antibody specificity [32].

To measure changes in oxytocin and CRF expressing cells in the whole paraventricular nucleus of the hypothalamus (PVN), three sections of the PVN (−1.5, −1.8, and −2.1 mm from bregma; Paxinos and Watson, 2004) per rat were imaged using a fluorescence microscope (10x objective, Axioimager Z3, Carl Zeiss, Germany) running ZenBlue software (Carl Zeiss). Oxytocin and CRF PVN cells across both hemispheres and from each bregma coordinate were quantified in each image using the cell counter plugin in ImageJ (National Institutes of Health, USA), with experimenters blind to experimental conditions.

### Statistical analysis

A power analysis was undertaken (G*Power 3.1.9.6, Faul et al., 2007) to determine condition sizes sufficient for detecting a moderate effect size with >80% power. Statistical analyses were performed using SPSS (v27, Mac; α = 0.05). Data were predominantly analysed using three-factor ANOVAs, with the between-subjects factors of sex, MS and adolescent treatment. Paired samples t-tests or mixed model ANOVAs were performed when the within-subjects factor of day was included. When the assumption of equal variance was not met, Greenhouse-Geisser corrections are reported. If a main effect or interaction of sex or priming dose was identified, males and females were analysed separately using 2 × 2 ANOVAs (MS×adolescent treatment). Litter was included as a covariate.

## Results

**For a more detailed results section, see the supplementary document**. For a summary of study findings, see Table 1.

**Table 1:** Summary of experiment 1 and 2 data. Arrows depict direction of change of significant main effects or interactions.

	Males		Females	
	Effect of Maternal Separation (MS360 relative to MS15)	Effect of oxytocin on maternal separation	Effect of Maternal Separation	Effect of oxytocin on maternal separation
*Anxiety*				
Time in Closed Arm on EPM	↑	↓	↑	↓
*Social Behaviour*				
Social Interaction (General Investigation)	–	–	↑	–
Social Interaction (Anogenital Sniffing)	↑	–	↑	–
Social Interaction (Aggressive behaviour)	↑	–	↑	–
*Methamphetamine addiction outcomes*				
Methamphetamine self-administration (0.03 mg/kg)	↑	–	–	–
Methamphetamine self-administration (0.1 mg/kg)	↑	↓	–	–
Lifetime methamphetamine intake (mg/kg)	↑	↓	–	–
Days to extinction criterion	↑	↓	–	–
Cue-induced reinstatement	↑	–	–	–
Meth-primed reinstatement (0.3 mg/kg)	–	-	-	-
Meth-primed reinstatement (1 mg/kg)	↑	↓	↑	↓
Yohimbine-primed reinstatement (0.625 mg/kg)	–	–	↑	↓
Yohimbine-primed reinstatement (1.25 mg/kg)	↑	↓	↑	–
*Brain and blood markers*				
Hypothalamic oxytocin cell counts	↓	↑	↓	–
Hypothalamic CRF cell counts	↑	–	↑	–
Plasma oxytocin levels	↑	↓	–	–
Plasma corticosterone levels	↑	↓	↑	↓

## Experiment 1

### Elevated Plus Maze

There was no significant sex effect on time spent in closed arms of the EPM. With sexes combined, the MS360 oxytocin treated (MS360-Oxytocin) rats spent less time in the closed arms relative to the MS360 vehicle-treated (MS360-Vehicle) rats, which differed to MS15 controls, where MS15 oxytocin (MS15-Oxytocin) and vehicle (MS15-Vehicle) rats spent a similar amount of time in the closed arms (interaction: F(1,97) = 7.740, *p* = 0.006; Fig. 1b). Additionally, there was a significant main effect of oxytocin treatment (F(1,97) = 12.844, *p* < 0.001), as oxytocin treated animals spent less time in the closed arms than vehicle treated rats.

### Social interaction

Time spent engaging in aggressive behaviours did not differ by sex. MS360 rats engaged in more aggressive behaviours than MS15 rats (F(1,72) = 4.389, p = 0.040; Fig. 1c). Oxytocin treatment did not affect aggressive behaviour (F(1,72) = 0.601, *p* = 0.441; interaction: F(1,72) = 1.504, *p* = 0.224).

## Methamphetamine IVSA

### Acquisition and maintenance

**Fig. 2:**
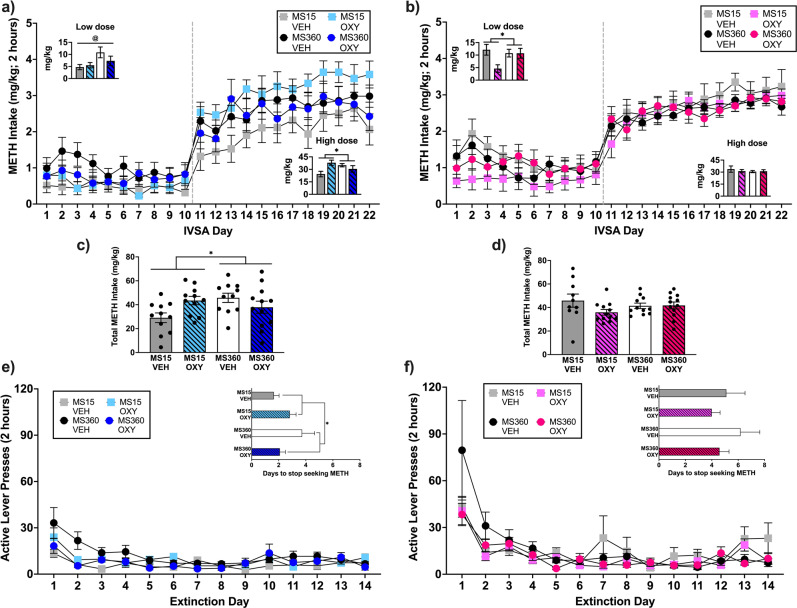
Methamphetamine intake across the 22-day IVSA period. **a** males and **b** females had access to a low METH dose (0.03 mg/kg/infusion) for the first 10 days, which was increased to the high dose (0.1 mg/kg/infusion) for the remaining 12 days. The inset graphs show total METH intake across MS and treatment conditions for each METH dose and total METH intake is depicted in **c** for males and **d** for females. Lever pressing during the daily extinction sessions for **e** male and **f** female rats. Inset graphs depict the number of extinction sessions required until rats reached the extinction criterion. Data are shown as mean ± SEM. *p < 0.05 significant interaction effect. OXY = Oxytocin, VEH = Vehicle. Sample size (*N* = 112) was reduced by the removal of 22 rats due to loss of catheter patency (*n* = 4 males), for not discriminating between the active and inactive lever (did not have a ratio of active to inactive lever presses of 2:1 per session for the last three acquisition sessions), or for not acquiring METH IVSA (did not take greater than 10 infusions per session over the last three acquisition sessions; male: *n* = 9, MS15 OXY = 4, MS360 VEH = 3, MS360 OXY = 2; female: *n* = 9, MS15 OXY = 5, MS360 VEH = 1, MS360 OXY = 3). This resulted in a sample size of 90 rats (males = 45, females = 45) with 10–13/condition/sex.

Rats increased their METH intake from day 1 to day 22 (F(7.488, 606.545) = 22.125, *p* < 0.005; Fig. 2a, b), and differentiated the active from the inactive lever (F(1,81) = 67.083, *p* < 0.001).

When the low METH dose was available, females self-administered more METH than males (*p* = 0.033). For males, MS360 rats self-administered more METH than MS15 rats (F(1,40) = 5.252, *p* = 0.027), however intake was not affected by adolescent oxytocin treatment (F(1,40) = 0.651, *p* = 0.425; interaction: F(1,40) = 1.595, *p* = 0.214). For females, adolescent oxytocin treatment reduced METH intake compared to adolescent vehicle treatment in MS15 rats, while METH intake was similar in MS360-Oxytocin and MS360-Vehicle rats (interaction: F(1,40) = 4.454, *p* = 0.041). A significant main effect of oxytocin treatment was also identified and largely driven by the MS15-Oxytocin condition (F(1,40) = 4.862, *p* = 0.033).

When the high METH dose was available, intake differed by sex, dependent on their adolescent treatment and ELS condition (sex × MS × treatment interaction: *p* = 0.005). For males, MS15-Oxytocin rats self-administered more METH than MS15-Vehicle rats, while MS360-Oxytocin rats self-administered less METH than MS360-Vehicle rats (interaction: F(1,40) = 10.067, *p* = 0.003). For females, METH intake did not differ by MS (F(1,40) = 0.367, *p* = 0.548) or by oxytocin treatment (F(1,40) = 0.283, *p* = 0.598; interaction: F(1,40) = 0.397, *p* = 0.532).

### Total METH intake

Total METH intake differed by sex, adolescent treatment, and MS exposure (sex×MS×adolescent-treatment interaction: *p* = 0.001). For males, MS15-Oxytocin rats self-administered more METH than MS15-Vehicle rats, while MS360-Oxytocin rats self-administered less METH than MS360-Vehicle rats (interaction: F(1,40) = 8.346, *p* = 0.006; Fig. 2c). For females, intake did not differ by MS (F(1,40) = 0.125, *p* = 0.726) or by oxytocin treatment (F(1,40) = 2.509, *p* = 0.121; interaction: F(1,40) = 2.566, *p* = 0.117; Fig. 2d).

### Extinction

Rats extinguished their active lever pressing (main effect of day: F(2.612, 211.550) = 23.751, *p* < 0.005; Fig. 2e, f). Females took longer to extinguish than males (*p* < 0.001). For males, MS360-Oxytocin rats extinguished faster than MS360-Vehicle rats, while MS15-Oxytocin rats took longer to extinguish than MS15-Vehicle rats (interaction: F(1,40) = 4.951, *p* = 0.032). Females reached the extinction criterion within a similar number of sessions regardless of MS (F(1,40) = 0.724, *p* = 0.400) or oxytocin treatment (F(1,40) = 1.721, *p* = 0.197; interaction: F(1,40) = 0.054, *p* = 0.817).

### Cue-induced reinstatement

**Fig. 3:**
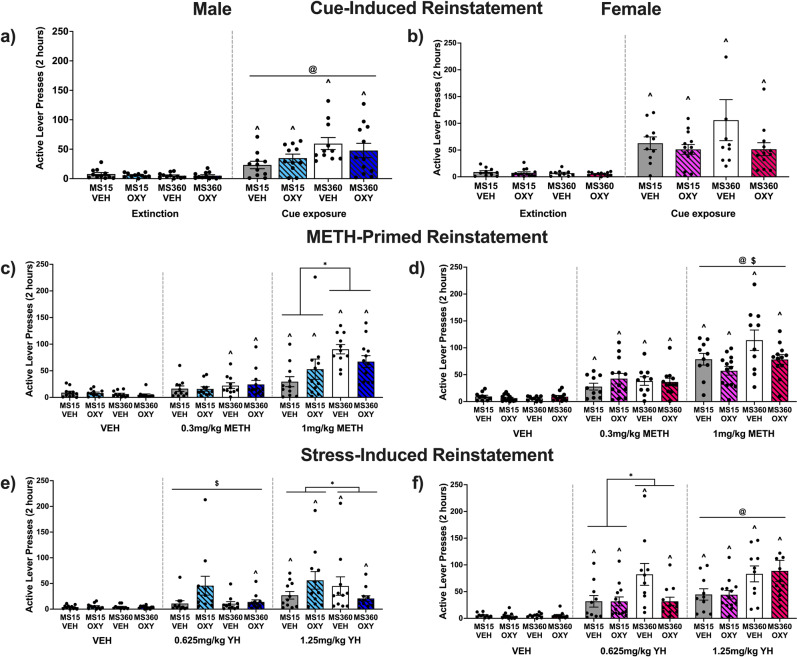
The effect of early life stress and adolescent oxytocin treatment on cue-, METH-, and yohimbine-induced reinstatement of drug-seeking. In males, early life stress increased cue- and METH (1 mg/kg) -induced reinstatement, which was reduced by oxytocin treatment in maternally separated rats. For females, early life stress increased METH (1 mg/kg)- and stress (yohimbine hydrochloride (yh) 0.625 mg/kg dose)-induced reinstatement, which was reduced by adolescent oxytocin treatment (*n* = 10–13/condition/sex). Mean (±SEM) active lever presses on cue-induced reinstatement (**a**) and (**b**), METH-primed reinstatement (**c**) and (**d**), and yohimbine-induced reinstatement (**e**) and (**f**) for males and females. VEH = Vehicle, OXY = Oxytocin. *p < 0.05 significant interaction effect, ^$^*p* < 0.05 significant adolescent treatment effect, @*p* < 0.05 significant MS effect ^*p* < 0.05 vs respective extinction or vehicle session.

All groups reinstated METH-seeking following cue re-exposure, compared to the extinction day prior (all paired t-tests *p* < 0.05). Females exhibited greater cue-induced active lever pressing than males (*p* = 0.016). Subsequent analyses in males revealed that active lever pressing was higher in MS360 rats relative to MS15 (F(1,40) = 6.068, *p* = 0.018; Fig. 3a). Active lever pressing did not differ by oxytocin treatment (F(1,40) = 0.000, *p* = 0.994; interaction: F(1,40) = 1.468, *p* = 0.233). For females, cue-induced active lever pressing did not differ by MS (F(1,40) = 1.341, *p* = 0.254) or oxytocin treatment (F(1,40) = 2.816, *p* = 0.101; interaction: F(1,40) = 1.201, *p* = 0.280; Fig. 3b).

### Methamphetamine-primed reinstatement

Active lever pressing during METH-primed reinstatement tests significantly differed by dose (F(2,161) = 48.585, *p* < 0.005) and sex (*p* = 0.001), and the interaction of sex x dose (*p* = 0.010). As such, subsequent analyses were conducted for each dose and sex.

#### 0.3 mg/kg METH

For males, compared to vehicle, 0.3 mg/kg METH significantly increased active lever pressing in MS360 rats (paired *t*-tests, *p* < 0.05), but not in MS15 rats (paired *t*-tests, *p* > 0.05; Fig. 3c). Lever pressing induced by this low METH dose was not significantly affected by MS (F(1,40) = 0.833, *p* = 0.367) or oxytocin treatment (F(1,40) = 0.067, *p* = 0.796; interaction: F(1,40) = 0.134, *p* = 0.716). Females in all conditions reinstated to this low METH dose (paired *t*-tests, *p* < 0.05; Fig. 3d), which was unaffected by MS (F(1,40) = 0.017, *p* = 0.898) or oxytocin treatment (F(1,40) = 0.715, *p* = 0.403; interaction: F(1,40) = 0.941, *p* = 0.338).

#### 1 mg/kg METH

Males reinstated following the high METH dose (paired *t*-tests, *p* < 0.05), and this active lever pressing was higher in MS360 rats than MS15 rats (main effect of MS: F(1,40) = 11.025, *p* = 0.002). Additionally, MS360-Oxytocin rats made fewer active lever presses than MS360-Vehicle males, whereas MS15-Oxytocin rats had higher lever pressing than MS15-Vehicle rats (interaction: F(1,40) = 4.245, *p* = 0.046). Females also reinstated to the high METH dose, compared with vehicle (paired *t*-tests, *p* < 0.05), and this was greater in MS360 than MS15 rats (F(1,40) = 6.543, *p* = 0.014). Additionally, oxytocin treated females made fewer active presses than vehicle treated females (F(1,40) = 6.262, *p* = 0.017). No interaction of MS × treatment was evident (F(1,40) = 0.442, *p* = 0.510).

### Yohimbine-induced reinstatement

Active lever pressing during yohimbine-induced reinstatement tests significantly differed by yohimbine dose (F(1.663,134.731) = 18.716, *p* < 0.005) and sex (main effect: *p* < 0.005; sex × dose interaction: *p* = 0.007). As such, subsequent analyses were conducted for each dose and sex.

#### 0.625 mg/kg yohimbine

For males, when compared to vehicle, 0.625 mg/kg yohimbine increased active lever pressing only in MS360-Oxytocin rats (paired *t*-test, *p* < 0.05; Fig. 3e). When comparing the effects of 0.625 mg/kg yohimbine between groups, oxytocin treated rats made more active lever presses than vehicle treated rats (F(1,40) = 4.113, *p* = 0.049), but there was no effect of MS (F(1,40) = 3.186, *p* = 0.082; interaction: F(1,40) = 2.169, *p* = 0.149).

In females, 0.625 mg/kg yohimbine increased active lever pressing compared to vehicle (paired *t*-tests, *p* < 0.05; Fig. 3f). In the test session, adolescent oxytocin treatment reduced lever pressing relative to vehicle treatment in MS360, but not MS15 rats (interaction: F(1,40) = 4.210, *p* = 0.047). The main effects of MS (F(1,40) = 4.435, *p* = 0.042) and oxytocin treatment (F(1,40) = 4.210, *p* = 0.047) were also significant.

#### 1.25 mg/kg yohimbine

Males in all conditions reinstated after 1.25 mg/kg yohimbine compared to vehicle (paired *t*-tests, *p* < 0.05). Adolescent oxytocin treatment reduced lever pressing in MS360 rats relative to vehicle treatment, while active lever pressing was higher in MS15-Oxytocin relative to MS15-Vehicle rats (interaction: F(1,40) = 6.125, *p* = 0.018).

Females in all conditions reinstated their drug-seeking behaviour (paired *t*-tests, *p* < 0.05). Active lever pressing was higher in MS360 compared to MS15 rats (F(1,40) = 8.806, *p* = 0.005), but was unaffected by oxytocin treatment (F(1,40) = 0.042, *p* = 0.839); interaction (F(1,40) = 0.051, *p* = 0.823).

## Experiment 2

### Oxytocin and CRF positive cells in the PVN

**Fig. 4:**
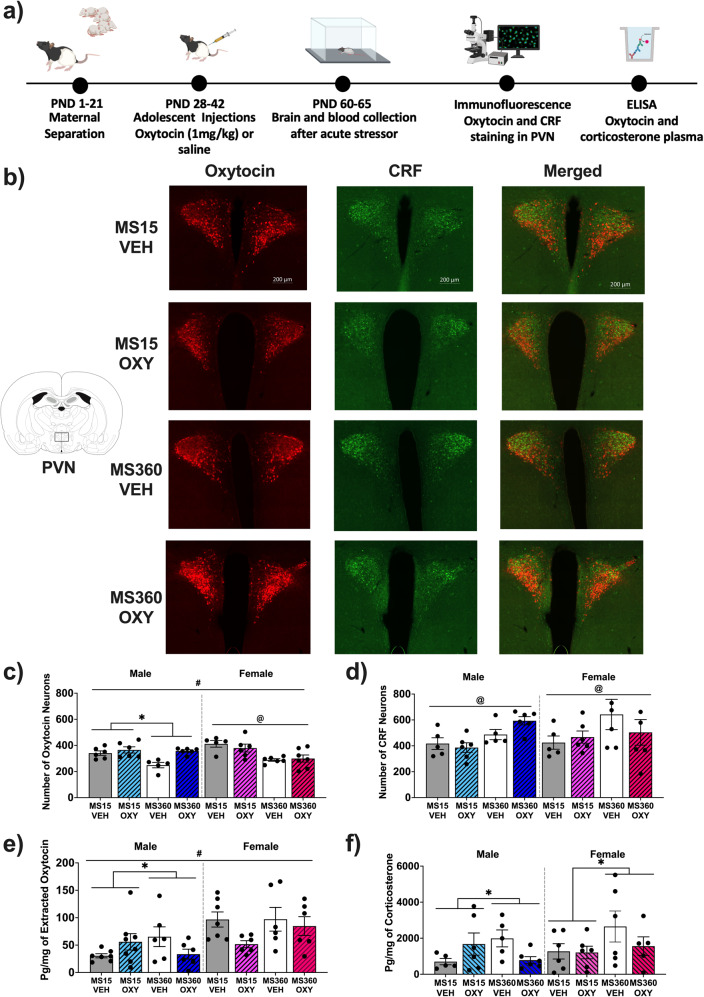
Effect of early life stress and adolescent oxytocin treatment on hypothalamic oxytocin and CRF neuronal expression. **a** experimental timeline depicting blood and brain analysis in malae and female Long Evan rats after ELS and adolescent oxytocin injections. Figure created in Biorender (academic subscription). Oxytocin and CRF neuronal staining in the PVN of the hypothalamus (−1.8 mm from Bregma) from a representative male from each MS and adolescent oxytocin treatment condition. **b** Images were taken at 20x magnification and have been adjusted for presentation purposes. Mean number (±SEM) in each condition of (**c**) oxytocin (*n* = 5–6/condition/sex) and (**d**) CRF positive neurons (*n* = 5–6/condition/sex) and (**e**) circulating oxytocin (*n* = 6–8/condition/sex) and (**f**) corticosterone plasma levels (*n* = 5–6/condition/sex). CRF corticotropin releasing factor, PVN paraventricular nucleus of the hypothalamus, VEH Vehicle, OXY Oxytocin *p < 0.05 significant interaction effect, ^#^*p* < 0.05 significant sex effect, ^@^*p* < 0.05 significant MS effect.

Expression of oxytocin positive neurons differed by sex and adolescent treatment (sex x adolescent treatment interaction; *p* = 0.024). For males, MS360-Oxytocin rats had more oxytocin positive cells than MS360-Vehicle rats, which differed from the MS15 conditions, where MS15-Vehicle and MS15-Oxytocin rats had similar numbers (interaction: F(1,18) = 4.894, *p* = 0.040; Fig. 4c). Significant main effects of MS (F(1,18) = 5.971, *p* = 0.025) and oxytocin treatment (F(1,18) = 12.302, *p* = 0.003) were also apparent. For females, MS360 rats had fewer oxytocin positive cells than MS15 rats (F(1,19) = 14.850, *p* = 0.001), however adolescent treatment did not impact numbers (F(1,19) = 0.069, *p* = 0.795; interaction: F(1,19) = 0.522, *p* = 0.479).

For CRF-positive cells, no sex differences were evident. With sexes combined, MS360 rats had more CRF positive cells than MS15 rats (F(1, 36) = 6.749, *p* = 0.014; Fig. 4d). However, adolescent oxytocin treatment did not change CRF numbers.

### Stress-induced oxytocin and corticosterone plasma concentration

Plasma oxytocin levels were higher in females relative to males (*p* < 0.05). For males, MS360-Oxytocin rats had lower oxytocin concentrations than MS360-Vehicle rats, which contrasted to MS15 rats, where oxytocin treated rats had higher concentrations than vehicle treated animals after the acute stressor (interaction: F(1,22) = 4.885, *p* = 0.038; Fig. 4e). For females, there were no significant differences in oxytocin concentrations across conditions (main effect of MS: F(1,20) = 0.843, *p* = 0.369; main effect of treatment: F(1,20) = 3.395, *p* = 0.080; interaction: F(1,20) = 1.209, *p* = 0.285).

Analysis of corticosterone concentrations in plasma revealed no significant difference between males and females. With sexes combined, corticosterone concentrations were lower in MS360-Oxytocin relative to MS360-Vehicle rats, which differed to the MS15 conditions, where corticosterone concentrations were similar across MS15-Vehicle and MS15-Oxytocin rats after the acute stressor (interaction: F(1,36) = 4.691, *p* = 0.037; Fig. 4f).

## Discussion

We determined whether oxytocin treatment during adolescence can ameliorate the effects of ELS on later-life anxiety, sociability, and addiction-relevant behaviours in male and female rats. We also investigated changes to the endogenous oxytocin and endocrine stress systems, as potential biological mediators between ELS, oxytocin treatment, and adverse outcomes. We discovered that, in both sexes, ELS increased anxiety-like behaviour and aggressive behaviours in adulthood, and that the emergence of elevated anxiety, but not aggressive behaviour, was prevented by adolescent oxytocin treatment. Furthermore, ELS exacerbated many facets of METH-seeking behaviour, with several notable sex differences emerging. Most strikingly, adolescent oxytocin treatment in males prevented ELS-enhancement of METH IVSA, perseverance during extinction, and METH-primed and yohimbine-induced reinstatement of drug seeking. In contrast, adolescent oxytocin treatment in females prevented the effects of ELS only on METH- and yohimbine-primed reinstatement. Accompanying the behavioural changes, we discovered that in males, ELS reduced the number of hypothalamic oxytocin-positive cells and, after an acute stressor, ELS was associated with increased circulating oxytocin levels, both of which were prevented by adolescent oxytocin treatment. ELS was also associated with increased hypothalamic CRF cell numbers and circulating corticosterone, and adolescent oxytocin treatment prevented the latter effects in both sexes. Taken together these findings support three claims. First, that ELS increases the risk of later vulnerability to drug-seeking behaviours differently between sexes. Second, adolescent oxytocin treatment can prevent the emergence of ELS-induced anxiety and addiction vulnerability in both sexes during adulthood. Lastly, that adolescent oxytocin treatment may prevent the behavioural effects of ELS from emerging by rehabilitating hypothalamic oxytocin neurons and corticosterone for males, and corticosterone for females, although these interactions need to be causally tested.

### Early life stress-induced sex-dependent phenotypic effects

ELS has a detrimental impact on mental health. Our results align with reports of MS increasing anxiety-like behaviour [33–35], social aggression [36], and the association between low PVN oxytocin cells and increased aggression in males and females [37]. Further, our data are consistent with reports of MS increasing PVN CRF-expressing cells [38–40] and reactive corticosterone plasma levels [41, 42] in both sexes. While previous studies have discovered changes in PVN oxytocin-expressing cells in MS-exposed adolescent males [7, 39, 43] and lactating females [44], our study is the first to demonstrate a reduction in PVN oxytocin cells in MS-exposed *adult* males and *non-lactating* females. Additionally, our discovery that oxytocin plasma release to stress is sensitised in MS-exposed males supports previous findings that in non-ELS animals, acute stress exposure increases circulating oxytocin concentrations [45]. These data complement previous demonstrations that MS dysregulates oxytocin receptor expression in rodents and that baseline oxytocin concentrations are lowered in the blood and cerebrospinal fluid of adults who experienced childhood trauma [46–49].

Unlike depression and anxiety, few studies have investigated vulnerability to stimulant addiction after ELS. In those that have, increased drug intake is typically reported in stressed males ([50–52] although see [53]), consistent with our findings here involving two different doses of METH. However, in contrast to our findings, others have not found enhanced cue-induced [50, 53] or drug primed-reinstatement [51]. This difference may be related to differences with the MS procedure or the drug studied (cocaine). Additionally, our study is the first to investigate yohimbine-induced reinstatement after ELS. We tested two doses of the pharmacological stressor yohimbine [26], to determine whether ELS increased sensitivity to yohimbine-induced reinstatement. Indeed, we found that females with a history of ELS were more sensitive to both doses of yohimbine than non-ELS females, while for males, ELS enhanced yohimbine reinstatement only at the higher dose. Although yohimbine was used presently as a model of stress-induced reinstatement based on its stress-inducing effects in humans [25], there is recent evidence to suggest that yohimbine may promote reinstatement by invigorating responding for cues [54]. Therefore, it is possible that the female rats in our study were more sensitive to yohimbine enhancement of cue salience or valence than males, rather than its stressor effects per se. This is consistent with human clinical data where yohimbine potentiated the effects of cue exposure on both craving and anxiety to a greater extent in women than in men [25], suggesting similarity of this sex and yohimbine interaction across species. Overall, however, our data demonstrate that stress exposure during a critical developmental period produces vulnerability to addiction, including drug intake and reinstatement, and that the sexes are differentially impacted.

It is worth noting that MS is one of the two most commonly implemented ELS procedures, the other being the limited bedding and nesting (LBN) model. The impact of MS, which mimics maternal neglect, on anxiety and drug-self administration in males and females may differ from the LBN model that mimics limited access to resources and severely adversely impacts the quality of maternal care [55]. Specifically, a shorter MS procedure increases anxiety-like behaviour in males relative to females, while females appear more affected after the LBN procedure [56]. In the only published study to date investigating psychostimulant self-administration after LBN, no difference in cocaine intake, motivation to take cocaine, or cue- and cocaine-primed reinstatement was identified in males exposed to the LBN procedure relative to controls [57]. In studies investigating opioid self-administration after ELS, exposure to the LBN procedure resulted in reduced morphine intake in males relative to controls [58]. This contrasted with females exposed to LBN who demonstrated increased resistance to extinction of drug-seeking, increased cue- and heroin-primed reinstatement, and increased opioid economic demand relative to controls [59]. Investigation into the potential differential effects of the LBN procedure on METH intake, extinction and relapse in both sexes would be a logical next step for understanding how different modes of ELS impact on the abuse potential of various drug classes across sexes.

### Adolescent oxytocin treatment prevented the emergence of the effects of early life stress differently between sexes

We discovered that adolescent oxytocin treatment in both sexes prevented the ELS-enhancement of adulthood anxiety, restored reactive corticosterone concentrations, and reduced drug- and yohimbine-induced reinstatement. Additionally, oxytocin treatment in ELS-exposed males was associated with reduced METH-intake, resistance to extinction, and restoration of oxytocin cell numbers in the PVN; improvements that were not evident in ELS-exposed females. These findings extend our previous work demonstrating that adolescent oxytocin treatment prevents depression-like behaviour in ELS-exposed males and females [12] and others’ findings that oxytocin treatment during early adolescence prevents anxiety-like behaviour in mid-adolescent male rats exposed to ELS [60]. Altogether, these findings suggest that the impact of ELS on brain systems which promote anxiety and addiction in adulthood may be buffered by adolescent oxytocin treatment, although not identically between sexes.

### Restoring the endogenous oxytocin system with exogenous oxytocin

Substance use disorders are associated with an altered endogenous oxytocin system. In post-mortem tissue from alcoholic males, PVN oxytocin-expressing cells were reduced [61], and in heroin-dependent individuals, oxytocin plasma concentrations negatively correlated with craving severity [62]. Similarly, oxytocin plasma and cerebrospinal fluid levels are lower in children who experienced trauma, and in adults with a history of ELS [46–48, 63, 64]. Through the benefit of animal models, we demonstrate that in males, ELS disrupts the oxytocin system and increases addiction-like behaviours; and crucially, that adolescent oxytocin treatment ameliorates this ELS-induced disruption to the endogenous oxytocin system, which is accompanied by a reduction in addiction-like behaviours. In contrast, although ELS increased addiction-like symptoms in females, and oxytocin treatment prevented this vulnerability, the ELS-induced disruption to PVN oxytocin-expressing cells was not restored by adolescent oxytocin treatment, and peripheral oxytocin levels were unaffected by ELS or oxytocin treatment. It is possible that exogenous oxytocin restored functioning of the oxytocin system in females in ways that we did not investigate, such as changes in oxytocin receptor expression or oxytocin neuronal firing properties. It should also be noted that we did not investigate changes to the PVN-OT system across the entire rostro-caudal and medio-lateral span, which may have provided better insight into the causes and consequences of these changes. Future studies measuring functional changes to the oxytocin system may provide insights into this sex-divergent finding. Together these data strongly support the theory that, at least in males, a perturbed oxytocin system arising from ELS, can increase addiction risk, and that this system may be rehabilitated using exogenous oxytocin, to reduce addiction vulnerability.

### Why might the efficacy of oxytocin treatment following early life stress depend upon sex?

Here we describe several instances of adolescent oxytocin treatment preventing the emergence of ELS-induced addiction vulnerability differentially between the sexes. Sex differences in oxytocin effects have been reported in various rodent models, including self-administration studies [65–67]. The underlying mechanisms are unclear with sex-dependent neurobiological effects of oxytocin only starting to be described in the literature. For example, Logan et al. [68] recently reported that oxytocin modulates nucleus accumbens (NAc) dopamine in cocaine-experienced male and female rats, but only modulates glutamate release in males. Additionally, expression of the oxytocin receptor differs according to sex with adult male rats having higher expression in the NAc, for example [69]. There is evidence that NAc oxytocin receptor activation can suppress drug-seeking behaviour in both sexes [70], although comprehensive analysis of such effects across sexes are yet to be conducted. Whether oxytocin engages other brain sites, and whether ELS alters oxytocin receptor expression differentially between the sexes, is currently unknown.

The oxytocin system develops differently between sexes during perinatal and early life periods. For example, the oxytocin peptide emerges in the brain of female rodents earlier than in males [71] and the female rodent brain exhibits greater oxytocin receptor expression throughout most of prenatal development [72]. Therefore, male and female rodents likely have different windows of developmental sensitivity for the oxytocin system, and our present use of the same PND range for oxytocin treatment in male and female rats did not account for this. Further research into the most critical developmental stage for targeting the oxytocin system across sexes would be useful.

It remains possible that the MS model of ELS may impact the oxytocin system to a greater extent in males than in females, while other models of ELS (e.g., LBN model), may differentially affect oxytocin system development. For example, the LBN procedure elicited earlier onset of puberty in female rats, while delaying puberty in male rats [73], and oxytocin receptor and oxytocin expression are increased in females, but decreased in males exposed to limited bedding [74].

Finally, our current findings of sex-dependent interactions of ELS and adolescent oxytocin treatment on stress-induced corticosterone release reflect our previous findings that adrenal weight is increased in female rats following MS. This increase in adrenal weight was prevented with adolescent oxytocin treatment, but male adrenals were unaffected by either manipulation [12]. It is therefore possible that adolescent oxytocin treatment lessens ELS-heightened corticosterone release in adulthood through distinct mechanisms in males and females. Indeed, oxytocin acts at the adrenal gland in humans to reduce cortisol levels [75] and adrenalectomy in male rats results in a diminished brain oxytocin response to an acute stressor [76]. Unfortunately, this bidirectional relationship between adrenal function and central oxytocin release has yet to be described in female rodents.

In summary, to shed better light on these interactions of sex, ELS and oxytocin treatment on addiction vulnerability, further studies utilizing different time windows for oxytocin intervention (e.g., shifted earlier in females), other ELS models (e.g., LBN) as well as more comprehensive neurobiological measures of oxytocin function (e.g., OTR binding, oxytocin neuron firing properties) and stress reactivity (e.g., adrenal function) would be welcome.

### Clinical implications

Our preclinical findings suggest there may be value in trialling oxytocin interventions in people who have experienced ELS. However, previous clinical experiments using intranasal oxytocin to treat neurodevelopmental and mental health disorders have produced mixed outcomes, which may dampen enthusiasm for oxytocin-based trials in clinical populations. One factor contributing to these disappointing clinical findings is insufficient power, whereby 73.5% of non-significant clinical findings were not sufficiently powered to detect a meaningful effect size [77]. In contrast, in sufficiently powered studies, oxytocin administration reduced craving for cocaine, opioids, and cannabis [78, 79]. Although no studies have investigated the efficacy of oxytocin for METH use disorder in people with confirmed disruptions to their oxytocin system, or with a history of ELS, our findings suggest that oxytocin could be a monotherapy or adjunctive pharmacotherapy for sufferers of ELS.

These findings, as well as previous findings by our group [12] and others [60] point to adolescence as an effective time point for initiating oxytocin treatment. Previous studies have shown that chronic oxytocin treatment in adulthood can reduce depression-like behaviour [11], so clinical treatment in adulthood may also be efficacious. However, adolescence is a critical neurodevelopmental window where the brain has the capacity to recalibrate if supported to do so with treatment [13]. Further, treatment during this period of maximal plasticity may be more likely to prevent the emergence of psychopathologies, and may treat them at an early stage. Importantly, clinical trials in children and young adolescents indicate that single and repeated oxytocin treatment is safe and tolerated at this age [80–82]. Lastly, the clinical efficacy of oxytocin may also depend on discovering how oxytocin interacts with sex hormones, menstrual phase, pregnancy, and the oral contraceptive pill, which all influence endogenous oxytocin [83–85].

## Conclusion

In summary, our findings link ELS with increased anxiety and addiction vulnerability, implicate the endogenous oxytocin system as a buffer between the two, and demonstrates that exogenous oxytocin can prevent some aspects of this ELS-induced psychopathology and oxytocin system perturbation. Given the clinical safety profile of oxytocin, and early signs of preclinical and clinical efficacy in treating substance use disorders, the further clinical investigation of adolescent oxytocin therapy for preventing ELS-associated mental health disorders seems warranted.

## Supplementary information

supplementary results
