# Supplementary material for: Oxytocin as an adolescent treatment for methamphetamine addiction after early life stress in male and female rats
Source: Neuropsychopharmacology. 2022 May 17;47(8):1561–73. doi: 10.1038/s41386-022-01336-y (PMC9206013; doi:10.1038/s41386-022-01336-y)
Supplement: Supplementary file 1 — supplementary results [file 41386_2022_1336_MOESM1_ESM.docx]

**Supplementary Results**

**Results**

**Experiment 1**

**Elevated Plus Maze**

Four samples (2 males, 2 females) were removed due to being outliers. No sex differences were identified (sex: F(1,97) = 0.047, p = 0.829, η_p_^2^ = 0.000; MS x sex (F(1,97) = 0.651, p = 0.422, η_p_^2^ = 0.007); treatment x sex (F(1,97) = 0.091, p = 0.764, η_p_^2^ = 0.001); MS x treatment x sex (F(1,97) = 1.155, p = 0.285, η_p_^2^ = 0.012)). With sexes combined, MS360 vehicle treated (MS360-Vehicle) rats spent an increased percentage of time in the closed arms relative to the MS360 oxytocin treated (MS360-Oxytocin) rats, and this differed to the MS15 animals, where MS15 oxytocin (MS15-Oxytocin) and vehicle (MS15-Vehicle) rats spent a similar amount of time in the closed arms (interaction: F(1,97) = 7.740, p = 0.006, η_p_^2^ = 0.074; Figure 3). The main effect of treatment (F(1,97) = 12.844, p < 0.001, η_p_^2^ = 0.117) was significant, with oxytocin treated animals spending less time in the closed arms, and the main effect of MS did not reveal a significant difference (F(1,97) = 0.083, p = 0.774, η_p_^2^ = 0.001).

**Social Interaction**

Males and females spent a similar amount of time engaging in anogenital sniffing (sex: F(1,72) = 1.689, p = 0.198, η_p_^2^ = 0.023; sex x MS: F(1,72) = 1.370, p = 0.246, η_p_^2^ = 0.019; sex x treatment: F(1,72) = 0.093, p = 0.761, η_p_^2^ = 0.001; sex x MS x treatment: F(1,72) = 3.813, p = 0.055, η_p_^2^ = 0.050). With sexes combined, MS360 rats spent more time engaging in anogenital sniffing than MS15 rats (MS: F(1,72) = 4.124, p = 0.046, η_p_^2^ = 0.054; Figure S1a). The main effect of treatment (F(1,72) = 2.531, p = 0.116, η_p_^2^ = 0.034) and the MS x treatment interaction were not significant (F(1,72) = 0.124, p = 0.725, η_p_^2^ = 0.002).

Analysis of general investigation revealed a significant sex difference (F(1,72) = 13.178, p = 0.001, η_p_^2^ = 0.155; Figure S1b). For males, subsequent analyses did not reveal any significant main (MS: F(1,36) = 0.040, p = 0.842, η_p_^2^ = 0.001; treatment: F(1,36) = 0.001, p = 0.979, η_p_^2^ = 0.000) or interaction effects (MS x treatment: F(1,36) = 0.508, p = 0.481, η_p_^2^ = 0.014). For females, MS360 rats engaged in more general investigation of the novel conspecific than MS15 rats (F(1,36) = 7.890, p = 0.008, η_p_^2^ = 0.180). The main effect of treatment (F(1,36) = 0.488, p = 0.489, η_p_^2^ = 0.013) and the MS x treatment interaction were not significant (F(1,36) = 0.073, p = 0.789, η_p_^2^ = 0.002).

Time spent engaging in aggressive behaviours did not differ by sex (sex: F(1,72) = 1.673, p = 0.200, η_p_^2^ = 0.023; sex x MS: F(1,72) = 1.037, p = 0.312, η_p_^2^ = 0.014; sex x treatment: F(1,72) = 1.576, p = 0.213, η_p_^2^ = 0.021; sex x MS x treatment: F(1,72) = 0.355, p = 0.553, η_p_^2^ = 0.005). MS360 rats engaged in more aggressive behaviours than MS15 rats (F(1,72) = 4.194, p = 0.044, η_p_^2^ = 0.055; Figure 4). The main effect of treatment was not significant (F(1,72) = 0.540, p = 0.465, η_p_^2^ = 0.007), nor was the MS x treatment interaction (F(1,72) = 1.936, p = 0.168, η_p_^2^ = 0.026).


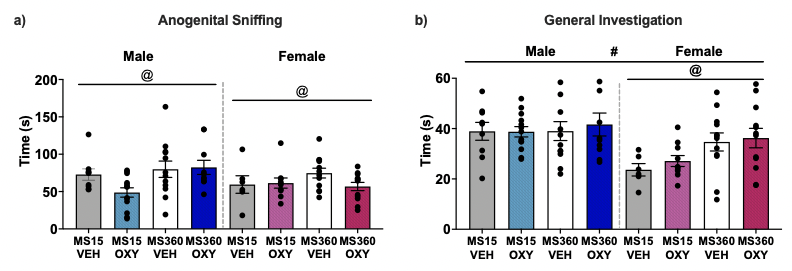


**Figure S1.** Maternal separation (MS360) changed how rats interacted with a novel conspecific relative to separation controls (MS15). Mean (± SEM) duration spent engaging in a) anogenital sniffing and b) general investigation by condition (n=6-12 pairs/condition/sex). VEH=Vehicle, OXY=Oxytocin. # p < 0.05 significant sex effect, @ p<0.05 significant MS effect.

**Methamphetamine IVSA**

Of the 112 rats, 22 rats were removed due to loss of catheter patency (n=4 males), for not discriminating between the active and inactive lever (did not have a ratio of active to inactive lever presses of 2:1 per session for the last three acquisition sessions) or for not acquiring meth IVSA (did not take greater than 10 infusions of METH over the last three days of acquisition; n=9 male, n=9 female). This resulted in a sample size of 45 males (MS15-Vehicle = 11, MS15-Oxytocin = 11, MS360-Vehicle = 11, MS360-Oxytocin = 12) and 45 females (MS15-Vehicle = 10, MS15-Oxytocin = 13, MS360-Vehicle= 10, MS360-Oxytocin = 12).

**Acquisition and maintenance**

Rats acquired meth IVSA. Rats increased their meth intake from day 1 to day 22 (day: F(7.488, 606.545) = 22.125, p < 0.005, η_p_^2^ = 0.215; Figures 5a and 5b), and differentiated the active from the inactive lever (lever: F(1,81)=67.083, p < 0.001, η_p_^2^ = 0.453).

Over the first 10 days of IVSA when the low meth dose was available, intake differed by sex (sex: F(1,81) = 4.683, p = 0.033, η_p_^2^ = 0.055; sex x MS x treatment interaction: F(1,81) = 6.106, p = 0.016, η_p_^2^ = 0.070). Similarly, intake also differed by sex when the high dose was available (sex x MS x treatment interaction: F(1,81) = 8.476, p = 0.005, η_p_^2^ = .095). As such, subsequent analyses were split by sex.

At the low dose, MS360 males took more meth than MS15 controls (MS: F(1,40) = 5.252, p = 0.027, η_p_^2^ = .116). No main effect of treatment (F(1,40) = 0.651, p = 0.425, η_p_^2^ = 0.016) or a MS x treatment interaction (F(1,40) = 1.595, p =0.214, η_p_^2^ = .038) were identified. Active lever pressing did not statistically differ by MS (F(1,40) = 3.915, p =0.055, η_p_^2^ = 0.089), treatment (F(1,40) = 0.742, p =0.394, η_p_^2^ = 0.018), or their interaction (F(1,40) = 1.605, p =0.213, η_p_^2^ = 0.039). Similarly, inactive lever pressing did not differ by MS (F(1,40) = 1.269, p =0.267, η_p_^2^ = 0.031), treatment (F(1,40) = 0.226, p =0.637, η_p_^2^ = 0.006) or by the MS x treatment interaction (F(1,40) = 1.467, p =0.233, η_p_^2^ = 0.035). For females, meth intake in the MS360-Oxytocin and MS360-Vehicle rats was similar, whilst in the MS15 conditions, MS15-Oxytocin rats self-administered less meth than the vehicle treated females (interaction: F(1,40) = 4.454, p = 0.041, η_p_^2^ = .100). A significant main effect of treatment was also identified and largely driven by the MS15- Oxytocin condition (F(1,40) = 4.862, p = 0.033, η_p_^2^ = .108). A main effect of MS was not evident (F(1,40) = 2.064, p =0.159, η_p_^2^ = .049). Similarly, for active lever pressing, a main effect of treatment was evident, largely driven by lower active lever pressing in the MS15-Oxytocin condition (F(1,40) = 4.360, p = 0.043). No main effect of MS was evident, nor was there a significant interaction. While inactive lever pressing was higher in MS360 rats relative to MS15 rats (F(1,40) = 6.560, p =0.014, η_p_^2^ = .141), inactive lever pressing did not differ by treatment (F(1,40) = 1.421, p =0.240, η_p_^2^ = .034) or by the MS x treatment interaction (F(1,40) = 1.167, p =0.286, η_p_^2^ = .028).

At the high dose, for males, MS15-Oxytocin males self-administered more meth than the MS15-Vehicle males, which differed to the MS360 condition where MS360-Oxytocin males self-administered less meth than MS360-Vehicle rats (interaction: F(1,40) = 10.067, p = 0.003, η_p_^2^ = .201). No significant main effects of MS (F(1,40) = 1.022, p = 0.318, η_p_^2^ = 0.025) or treatment (F(1,40) = 1.789, p =0.189, η_p_^2^ = 0.043) were evident. A similar pattern was evident for active lever pressing, whereby a significant MS x treatment interaction was evident (F(1,40) = 9.013, p =0.005, η_p_^2^ = .0184), and the main effects of MS (F(1,40) = 2.385, p =0.130, η_p_^2^ = .056) and treatment (F(1,40) = 1.929, p =0.173, η_p_^2^ = 0.046) were not significant. Inactive lever pressing did not differ by MS (F(1,40) = 0.841, p =0.365, η_p_^2^ = .021), treatment (F(1,40) = 0.478, p =0.493, η_p_^2^ = 0.012), or the MS x treatment interaction (F(1,40) = 0.847, p =0.363, η_p_^2^ = 0.021). For females, meth intake did not differ by MS (F(1,40) = 0.367, p = 0.548, η_p_^2^ = 0.009) or by treatment (F(1,40) = 0.283, p = 0.598, η_p_^2^ = .007), nor was there a significant interaction (F(1,40) = 0.397, p = 0.532, η_p_^2^ = 0.010). Similarly active lever pressing did not differ by MS (F(1,40) = 0.000, p =0.989, η_p_^2^ = 0.000), treatment (F(1,40) = 0.337, p =0.565, η_p_^2^ = 0.008), or their interaction (F(1,40) = 0.025, p =0.876, η_p_^2^ = 0.001). For inactive lever pressing, a significant interaction was evident, whereby inactive lever pressing was similar in MS360-Vehicle and MS360-Oxytocin rats, and in MS15 rats, MS15-Oxytocin rats had higher inactive pressing than MS15-Vehicle rats (F(1,40) = 6.933, p =0.012, η_p_^2^ = .0148). Inactive lever pressing differed by treatment (F(1,40) = 9.890, p =0.003, η_p_^2^ = .0198), but not by MS (F(1,40) = 0.402, p =0.530, η_p_^2^ = 0.010).

**Total meth intake**

Over the course of meth IVSA, intake differed by sex dependent on adolescent treatment and MS exposure (sex x MS x adolescent treatment interaction: F(1,81) = 10.913, p = 0.001, η_p_^2^ = .119). For males, similar to the high dose, MS15-Oxytocin rats took more meth than the MS15-Vehicle rats over the IVSA period, while MS360-Oxytocin rats took less meth than MS360-Vehicle rats (interaction: F(1,40) = 8.346, p = 0.006, η_p_^2^ = .173; Figure 5c). Main effects of MS (F(1,40) = 2.896, p = 0.097, η_p_^2^ = .068) and oxytocin treatment (F(1,40) = 0.441, p = 0.510, η_p_^2^ = .011) were not significant. For females, lifetime meth intake did not differ by MS (F(1,40) = 0.125, p = 0.726, η_p_^2^ = .003) or by oxytocin treatment (F(1,40) = 2.509, p = 0.121, η_p_^2^ = .059; interaction: F(1,40) = 2.566, p = 0.117, η_p_^2^ = .060; Figure 5d).


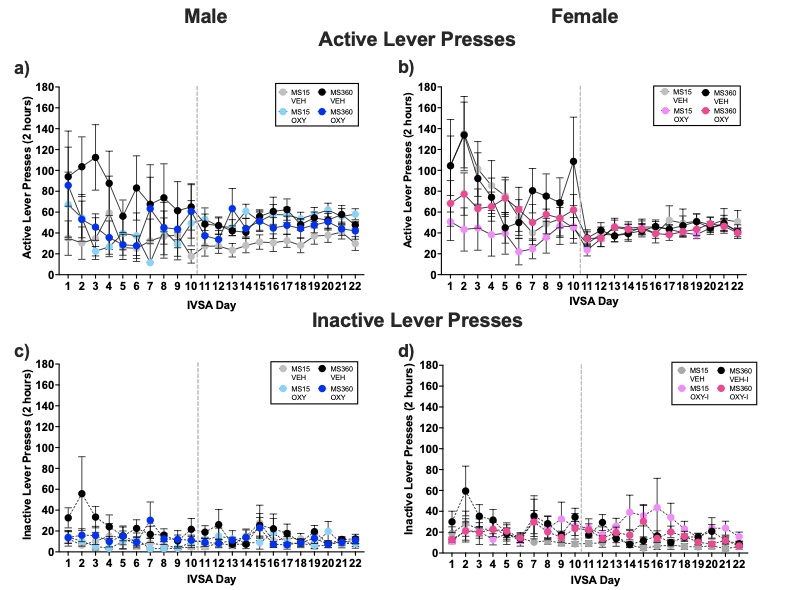


**Figure S2.** Mean (± SEM) number of active (a and b) and inactive lever presses (c and d) across the 22 day methamphetamine self-administration period for males and females. VEH=Vehicle, OXY=Oxytocin.

**Extinction**

All rats extinguished their active lever pressing (F(2.612, 211.550) = 23.751, p < 0.005, η_p_^2^ = 0.227; Figures 6a and 6b). Females took longer to extinguish than males (sex: F(1,81) = 15.521, p < 0.001, η_p_^2^ = 0.161). For males, MS360-Oxytocin rats extinguished faster than MS360-Vehicle rats, which differed to the MS15 rats, where MS15-Oxytocin males took longer to extinguish than MS15-Vehicle controls (interaction: F(1,40) = 4.951, p = 0.032, η_p_^2^ = 0.110). No significant main effects of MS (F(1,40) = 0.617, p < 0.437, η_p_^2^ = 0.015) or treatment were evident (F(1,40) = 0.088, p = 0.768, η_p_^2^ = 0.002). Females achieved extinction criterion within a similar number of extinction sessions regardless of MS (F(1,40) = 0.724, p =0.400, η_p_^2^ = 0.018) or treatment (F(1,40) = 1.721, p = 0.197, η_p_^2^ = 0.041). Additionally, the MS x treatment interaction was not significant (F(1,40) = 0.054, p = 0.817, η_p_^2^ = 0.001).

**Cue-Induced reinstatement**

All males and females demonstrated reinstatement of meth-seeking behaviour on cue exposure, as indicated by significantly higher active lever-pressing on the cue reinstatement test session relative to the extinction day prior across each condition (males: MS15-Vehicle t(10) -3.336, p = 0.008, MS15-Oxytocin t(10) -4.570, p = 0.001, MS360-Vehicle t(10) -5.642, p < 0.001, MS360-Oxytocin t(11) -3.452, p = 0.005; females: MS15-Vehicle t(9) -4.306, p = 0.002, MS15-Oxytocin t(12) -4.952, p < 0.001, MS360-Vehicle t(9) -2.669, p = 0.026, MS360-Oxytocin t(11) -3.758, p = 0.003). When comparing active lever pressing on the cue-relapse test session across conditions, a significant sex difference was revealed (F(1,81) = 6.086, p = 0.016, η_p_^2^ = 0.070). Subsequent analyses in males revealed that active lever pressing was higher in the MS360 condition relative to MS15 males (MS: F(1,40) = 6.068, p = 0.018, η_p_^2^ = 0.132; Figure 7a). No significant treatment effect (F(1,40) = 0.000, p = 0.994, η_p_^2^ = 0.000) or MS x treatment interaction were demonstrated (F(1,40) = 1.468, p = 0.233, η_p_^2^ = 0.035). A significant difference in inactive lever pressing based on MS condition was also evident (F(1,40) = 7.000, p = 0.012, η_p_^2^ = 0.149; see supplementary table 1), although inactive lever pressing across conditions was lower than 10 inactive lever presses and was significantly lower than active lever pressing (F(1,40) = 8.052, p = 0.007, η_p_^2^ = 0.168).

For females, no significant main effects of MS (F(1,40) = 1.341, p = 0.254, η_p_^2^ = 0.032) or treatment (F(1,40) = 2.816, p = 0.101, η_p_^2^ = 0.066) on active lever pressing were evident, nor was there a significant MS x treatment interaction (F(1,40) = 1.201, p = 0.280, η_p_^2^ = 0.029). Females did press the active lever across conditions significantly more than the inactive lever (F(1,40) = 7.519, p = 0.009, η_p_^2^ = 0.1158). When examining inactive lever pressing, no significant main effects of MS (F(1,40) = 1.795, p = 0.188, η_p_^2^ = 0.043) or treatment (F(1,40) = 0.333, p = 0.567, η_p_^2^ = 0.008) were apparent. A significant MS x treatment interaction effect was identified (F(1,40) = 5.887, p = 0.020, η_p_^2^ = 0.128) whereby inactive lever pressing was higher in the MS15-Oxytocin rats relative to the MS15-Vehicle rats, and this differed to the MS360 condition, where inactive lever pressing was lower in the MS360-Oxytocin rats relative to the MS360-Vehicle rats.

**Supplementary table 1:** Mean (SEM) inactive lever pressing across cue-, meth-, and yohimbine-induced reinstatement test sessions for each condition and sex.

**Methamphetamine-primed reinstatement**

Active lever pressing significantly differed by meth dose (dose: F(2,161) = 48.585, p < 0.005, η_p_^2^ = 0.375) and sex (sex: F(1,81) = 13.036, p = 0.001, η_p_^2^ = 0.139; sex x dose: F(2,162) = 4.753, p = 0.010, η_p_^2^ = 0.055). As such, subsequent analyses were conducted for each dose and sex.

***0.3mg/kg meth***

Relative to vehicle, active lever pressing increased after a low meth dose in males in MS360 conditions (vehicle: t(10) = -3.064, p = 0.012; oxytocin: t(11) = -2.702, p = 0.021), but not in MS15 conditions (vehicle: t(10) = -2.515, p = 0.057; oxytocin: t(10) = -2.128, p = 0.059) indicating that MS360 males reinstated to their prior drug-seeking behaviour (Figure 7c). Even so, lever pressing did not significantly differ on the 0.3 mg/kg test day across MS (F(1,40)= 0.833, p =0.367, η_p_^2^ = 0.020) or treatment conditions (F(1,40)= 0.067, p = 0.796, η_p_^2^ =0.002; interaction: F(1,40) = 0.134, p = 0.716, η_p_^2^ = 0.003). For females across all conditions, lever pressing was higher after a low meth dose relative to vehicle (MS15-Vehicle t(9) -2.655, p = 0.026, MS15-Oxytocin t(12) -3.646, p = 0.003, MS360-Vehicle t(9) -4.375, p = 0.002, MS360- Oxytocin t(11) -4.949, p < 0.001; Figure 7d). Even though all females reinstated, lever pressing did not differ across conditions (MS: F(1,40) = 0.017, p = 0.898, η_p_^2^ = 0.000; treatment: F(1,40) = 0.715, p = 0.403, η_p_^2^ = 0.018; interaction: F(1,40) = 0.941, p = 0.338, η_p_^2^ = 0.023). For both sexes, active and inactive lever pressing did not differ (males: F(1,40) = 0.010, p = 0.923, η_p_^2^ = 0.000; females: F(1,40) = 1.918, p = 0.174, η_p_^2^ = 0.047). Analysis of inactive pressing for males revealed a significant main effect of MS (F(1,40) = 7.646, p = 0.009, η_p_^2^ = 0.160) whereby inactive lever pressing was higher in MS360 rats relative to MS15 rats. Adolescent treatment did not significantly differ (F(1,40) = 0.441, p = 0.510, η_p_^2^ = 0.011) nor was there a significant MS x adolescent treatment interaction (F(1,40) = 1.144, p = 0.291, η_p_^2^ = 0.028). For females, inactive lever pressing did not differ by MS condition F(1,40) = 0.046, p = 0.831, η_p_^2^ = 0.001 or adolescent treatment F(1,40) = 3.172, p = 0.083, η_p_^2^ = 0.075), although a significant MS x adolescent treatment interaction was evident F(1,40) = 5.945, p = 0.019, η_p_^2^ = 0.132) whereby inactive pressing was similar across MS conditions, while for MS15 conditions, MS15-Vehicle rats had lower inactive presses relative to MS15-Oxytocin rats.

***1mg/kg meth***

Active lever pressing on the test session was significantly higher than vehicle for males in all 4 conditions (MS15-Vehicle t(10) -2.372, p = 0.039, MS15-Oxytocin t(10) -2.336, p = 0.042, MS360-Vehicle t(10) -10.504, p < 0.001, MS360-Oxytocin t(11) -5.584, p < 0.001). On the test session, MS360 rats pressed the active lever more than the MS15 rats (MS: F(1,40) = 11.025, p = 0.002, η_p_^2^ = 0.216). Additionally, MS360-Vehicle rats had higher active lever pressing than MS360-Oxytocin males, and this differed to MS15 rats, where MS15-Oxytocin rats had higher lever pressing than MS15-Vehicle rats (interaction: F(1,40) = 4.245, p = 0.046, η_p_^2^ = 0.096). The main effect of treatment was not significant (F(1,40) = 0.033, p = 0.858, η_p_^2^ = 0.001). When comparing active and inactive lever pressing, active lever pressing was significantly higher than inactive pressing across conditions (F(1,40) = 23.110, p < 0.005, η_p_^2^ = 0.366). Inactive pressing differed by MS condition, whereby inactive pressing, while significantly lower than active pressing, was higher in the MS360 condition relative to the MS15 condition (F(1,40) = 4.281, p = 0.045, η_p_^2^ = 0.097). Inactive pressing did not differ by adolescent treatment (F(1,40) = 0.000, p = 0.993, η_p_^2^ = 0.000), nor was there a significant MS x adolescent treatment interaction (F(1,40) = 0.074, p = 0.787, η_p_^2^ = 0.002).

For females, like males, active lever pressing following a 1 mg/kg meth priming injection was significantly higher than vehicle across all conditions (MS15-Vehicle t(9) -5.929, p < 0.001, MS15-Oxytocin t(12) -6.554, p < 0.001, MS360-Vehicle t(9) -5.864, p < 0.001, MS360-Oxytocin t(11) -8.007, p < 0.001). On the test session MS360 females pressed the active lever more than the MS15 rats (MS: F(1,40) = 6.543, p = 0.014, η_p_^2^ = 0.141). Additionally, a significant main effect of treatment was demonstrated (F(1,40) = 6.262, p = 0.017, η_p_^2^ = 0.135) where oxytocin treated females made fewer active presses than saline treated rats. No significant MS x treatment interaction was evident (F(1,40) = 0.442, p = 0.510, η_p_^2^ = 0.011). When comparing active and inactive pressing, active pressing was higher than inactive lever pressing (F(1,40) = 7.623, p = 0.009, η_p_^2^ = 0.164). Inactive lever pressing did not differ across MS condition (F(1,40) = 2.335, p = 0.135, η_p_^2^ = 0.056) or adolescent treatment (F(1,40) = 1.527, p = 0.224, η_p_^2^ = 0.038), nor was there a significant MS x adolescent interaction F(1,40) = 3.930, p = 0.054, η_p_^2^ = 0.092).

**yohimbine-primed reinstatement**

Active lever pressing significantly differed by yohimbine dose (dose: F(1.663,134.731) = 18.716, p < 0.005, η_p_^2^ = 0.188) and sex (sex: F(1,81) = 17.839, p < 0.005, η_p_^2^ = 0.180; sex x dose: F(1.663,134.731) = 5.117, p = 0.007, η_p_^2^ = 0.059). As such, subsequent analyses were conducted for each individual dose and for each sex.

***0.625mg/kg yohimbine***

Active lever pressing after a 0.625 mg/kg yohimbine injection did not significantly differ to vehicle in males from both MS15 conditions and the MS360-Vehicle condition (MS15-Vehicle t(9) -1.265, p = 0.235, MS15-Oxytocin t(12) -2.201, p = 0.052, MS360-Vehicle t(9) -1.501, p = 0.164), however, males in the MS360-Oxytocin pressed the active lever significantly more on the test session relative to vehicle (t(11) -2.473, p = 0.031; Figure 7e). On the test session, a significant treatment effect was evident (F(1,40) = 4.113, p = 0.049, η_p_^2^ = 0.093), whereby active pressing was higher in oxytocin-treated males. No main effect of MS (F(1,40) = 3.186, p = 0.082, η_p_^2^ = 0.074) or the interaction (F(1,40) = 2.169, p = 0.149, η_p_^2^ = 0.051) were significant. Inactive and active lever pressing significantly differed, whereby active pressing was higher than inactive pressing (F(1,40) = 6.905, p = 0.012, η_p_^2^ = 0.144). Inactive lever pressing did differ by treatment (F(1,40) = 4.623, p = 0.037, η_p_^2^ = 0.101) where oxytocin-treated animals pressed the inactive lever more than vehicle treated animals. Inactive pressing did not differ by MS condition (F(1,40) = 1.242, p = 0.272, η_p_^2^ =0.029), nor was there a significant MS x adolescent treatment interaction (F(1,40) = 2.706, p = 0.108, η_p_^2^ = 0.062).

Females in each condition reinstated to drug-seeking behaviour following a 0.625 mg/kg injection of yohimbine (MS15-Vehicle t(9) -3.743, p = 0.005, MS15-Oxytocin t(12) -5.122, p < 0.001, MS360-Vehicle t(9) -5.431, p < 0.001, MS360-Oxytocin t(11) -4.232, p = 0.001; Figure 7f). On the test session, active lever pressing was significantly lower in the MS360-Oxytocin rats relative to MS360-Vehicle females, which differed to the MS15 condition, where lever pressing was similar across adolescent treatment conditions (interaction: F(1,40) = 4.210, p = 0.047, η_p_^2^ = 0.095). The main effects of MS (F(1,40) = 4.435, p = 0.042, η_p_^2^ = 0.100) and treatment (F(1,40) = 4.210, p = 0.047, η_p_^2^ = 0.095) were also significant. Active and inactive lever pressing significantly differed, where active lever pressing was higher than inactive pressing (F(1,40) = 4.286, p = 0.045, η_p_^2^ = 0.095). Inactive pressing on the test session did differ, as evidenced by a significant MS x adolescent treatment interaction (F(1,40) = 5.424, p = 0.025, η_p_^2^ = 0.117) whereby MS360-Vehicle rats had higher lever pressing than MS360-Oxytocin rats which was in contrast to MS15 rats, where MS15-Oxytocin rats had higher lever pressing than MS15-Vehicle rats. A significant main effect of MS condition was evident (F(1,40) = 7.212, p = 0.010, η_p_^2^ = 0.150) and the main effect of adolescent treatment was not significant (F(1,40) = 1.072, p = 0.307, η_p_^2^ = 0.025).

***1.25mg/kg yohimbine***

For males in each condition, active lever pressing was significantly higher after the 1.25 mg/kg yohimbine injection compared to vehicle (MS15-Vehicle t(10) -3.332, p = 0.008, MS15-Oxytocin t(10) -2.917, p = 0.015, MS360-Vehicle t(10) -2.348, p = 0.041, MS360-Oxytocin t(11) -3.244, p = 0.008). On the test session, active lever pressing was lower in MS360-Oxytocin rats relative to MS360-Vehicle rats, and this differed to MS15 rats, where active lever pressing was higher in MS15-Oxytocin rats relative to MS15-Vehicle rats (interaction: F(1,40) = 6.125, p = 0.018, η_p_^2^ = 0.133). The main effects of MS (F(1,40) = 0.002, p = 0.967, η_p_^2^ = 0.000) and treatment (F(1,40) = 0.020, p = 0.889, η_p_^2^ = 0.000) were not significant. Active lever pressing was higher than inactive lever pressing on the test session (F(1,40) = 10.485, p = 0.002, η_p_^2^ = 0.208). Inactive lever pressing did not differ by MS condition (F(1,40) = 0.543, p = 0.466, η_p_^2^ = 0.013) or adolescent treatment (F(1,40) = 0.016, p = 0.900, η_p_^2^ = 0.000), nor was there a significant MS x adolescent treatment interaction (F(1,40) = 1.456, p = 0.235, η_p_^2^ = 0.035).

Females also reinstated to their prior drug-seeking behaviour (MS15-Vehicle t(9) -3.743, p = 0.005, MS15-Oxytocin t(12) -5.122, p < 0.001, MS360-Vehicle t(9) -5.431, p < 0.001, MS360-Oxytocin t(11) -4.232, p = 0.001). On the test session, active lever pressing was significantly higher in MS360 females relative to MS15 rats (F(1,40) = 8.806, p = 0.005, η_p_^2^ = 0.180). The main effect of treatment (F(1,40) = 0.042, p = 0.839, η_p_^2^ = 0.001) and the MS x treatment interaction (F(1,40) = 0.051, p = 0.823, η_p_^2^ = 0.001) were not significant. Active pressing did not differ to inactive lever pressing (F(1,40) = 1.091, p = 0.302, η_p_^2^ = 0.027). Inactive pressing on the test session did differ, as evidenced by a significant MS x adolescent treatment interaction (F(1,40) = 5.124, p = 0.029, η_p_^2^ = 0.114) whereby MS360-Vehicle rats had higher lever pressing than MS360-Oxytocin rats which was in contrast to MS15 rats, where MS15-Oxytocin rats had higher lever pressing than MS15-Vehicle rats. Inactive lever pressing did not differ by adolescent treatment (F(1,40) = 0.304, p = 0.584, η_p_^2^ = 0.008), or by MS condition (F(1,40) = 2.815, p = 0.101, η_p_^2^ = 0.066).

**Experiment 2**

***Oxytocin and CRF positive cells in the PVN***

For oxytocin positive cells, one way ANOVA showed a significant sex x adolescent treatment interaction (F(1, 38) = 5.514, p = 0.024, η_p_^2^ = 0.127). As such, subsequent analyses were conducted for each sex separately. For males, the MS360-Vehicle animals had fewer oxytocin positive cells than the MS360-Oxytocin condition, whilst oxytocin-labelled cells were similar across MS15 controls (interaction: F(1,18) = 4.894, p = 0.040, η_p_^2^ = 0.214; Figure 2b). Significant main effects of MS (F(1,18) = 5.971, p = 0.025, η_p_^2^ = 0.249) and treatment (F(1,18) = 12.302, p = 0.003, η_p_^2^ = 0.406) were also apparent. For females, MS360 animals had fewer oxytocin positive cells than MS15 controls (MS: F(1,19) = 14.850, p =0.001, η_p_^2^= 0.439). There was no significant main effect of treatment (F(1,19) = 0.069, p = 0.795, η_p_^2^ = 0.004) nor was there a significant interaction (F(1,19) = 0.522, p = 0.479, η_p_^2^ = 0.027).

For CRF positive cells, no sex differences were evident (sex: F(1, 36) = 0.153, p 0.698, η_p_^2^ = 0.004; sex x treatment: F(1, 36) = 0.551, p 0.463, η_p_^2^ = 0.015; sex x MS: F(1, 36) = 0.051, p 0.822, η_p_^2^ = 0.001; sex x MS x treatment interaction; F(1, 36) = 3.068, p 0.088, η_p_^2^ = 0.079). With sexes combined, MS360 animals had more CRF-positive cells than MS15 controls (MS: F(1, 36) = 6.749, p 0.014, η_p_^2^ = 0.158; Figure 2c). No significant treatment main effect (F(1, 36) = 0.018, p 0.895, η_p_^2^ = 0.000) or interaction (F(1, 36) = 0.110, p 0.742, η_p_^2^ = 0.003) were evident.

***Oxytocin and corticosterone immunoassays***

For the immunoassays, two male and one female samples were removed; two due to being outliers and one due to a sample error with the BCA analysis.

Analysis of oxytocin plasma revealed sex differences (sex: F(1,43) = 9.373, p = 0.004, η_p_^2^ = 0.179; MS x treatment x sex interaction: F(1,43) = 4.479, p = 0.040, η_p_^2^ = 0.094), resulting in subsequent analyses being conducted separately for each sex. For males, MS360-Vehicle males had higher oxytocin blood plasma levels than MS360-Oxytocin males and this differed to MS15 animals, where oxytocin treated animals had higher plasma levels than vehicle treated animals (interaction: F(1,22) = 4.885, p = 0.038, η_p_^2^ = 0.182; Figure 2d). None of the main effects were significant (MS: F(1,22) = 0.000, p = 0.990, η_p_^2^ = 0.000; treatment: F(1,22) = 0.011, p = 0.919, η_p_^2^ = 0.000). For females, no significant differences were identified (MS: F(1,20) = 0.843, p = 0.369, η_p_^2^ = 0.040; treatment: F(1,20) = 3.395, p = 0.080, η_p_^2^ = 0.145; interaction: (F(1,20) = 1.209, p = 0.285, η_p_^2^ = 0.057).

Analysis of corticosterone plasma revealed no significant difference between males and females (F(1,36) = 1.265, p = 0.268, η_p_^2^ = 0.034). With both sexes combined, corticosterone levels were higher in MS360-Vehicle animals relative to MS360-Oxytocin animals and circulating corticosterone levels were similar across MS15-Vehicle and MS15-Oxytocin animals (MS x treatment interaction: F(1,36) = 4.691, p = 0.037, η_p_^2^ = 0.115; Figure 2e). No other interactions (MS x sex (F(1,36) = 0.567, p = 0.457, η_p_^2^ = 0.015); treatment x sex (F(1,36) = 0.484, p = 0.491, η_p_^2^ = 0.013); MS x treatment x sex (F(1,36) = 0.691, p = 0.411, η_p_^2^ = 0.019)) or main effects (MS (F(1,36) = 2.156, p = 0.151, η_p_^2^ = 0.057); treatment (F1,36) = 1.025, p = 0.318, η_p_^2^ = 0.028)) were significant.
